# Prevalence and associated factors of depression, anxiety, and stress among pregnant women in Herat, Afghanistan: A cross‐sectional study

**DOI:** 10.1002/hsr2.1490

**Published:** 2023-08-11

**Authors:** Aziz‐ur‐Rahman Niazi, Mina Alekozay, Khadejah Osmani, Abdul Fattah Najm

**Affiliations:** ^1^ Department of Public Health and Infectious Diseases, Faculty of Medicine Herat University Herat Afghanistan; ^2^ Department of Surgery, Division of Obstetrics and Gynecology, Faculty of Medicine Herat University Herat Afghanistan; ^3^ Mental Health Program International Assistance Mission Herat Afghanistan

**Keywords:** Afghanistan, anxiety, depression, Herat, pregnant women, stress

## Abstract

**Background and Aims:**

Pregnancy is an important and natural event in a woman's life. It represents a time of substantial social and bio‐psychological challenges for a pregnant woman that may increase vulnerability to emotional disturbances such as depression, anxiety, and stress (DAS). This study aims to assess the prevalence and associated factors of DAS among pregnant women in Herat city of Afghanistan.

**Methods:**

This hospital‐based cross‐sectional study was conducted between July and November 2019, on 691 Dari‐speaking pregnant women aged 15‐49 years, who attended the antenatal clinic of Herat Razaei Maternity Hospital. The interview process involved the use of a structured questionnaire that collected data on sociodemographic characteristics of participants, as well as the validated Dari‐translated version of DASS‐42 questionnaire. A *χ* ^2^ test was used to test for association between categorical data. Forward likelihood ratio was used to assess the strength of association between sociodemographic variables and DAS; independently. The significance level was set to 0.05 and the confidence interval to 95% in all statistical analyses. Statistical analyses were performed in IBM SPSS Statistics (version 27).

**Results:**

The mean age of participants was 26.17 ± 6.06. The overall prevalence of DAS among study participants were 42.8%, 40.0%, and 59.5%, respectively. Planning of current pregnancy, women's health, husband's health, women's nutrition, family support, husband's support, women's education, women's employment, and family economy were significantly associated with DAS, while gestational age was not significantly associated with DAS.

**Conclusion:**

The prevalence of DAS among pregnant women in Herat city of Afghanistan is very high. Considering scientific evidence on high prevalence and associated factors of DAS among pregnant women, policymakers, public health authorities, and medical practitioners must devote significant attention in reducing the magnitude of these mental disorders and/or reducing their impact on women, their families, and the society.

## 1 INTRODUCTION

Pregnancy is an important and natural event in a woman's life. It represents a time of substantial social and bio‐psychological challenges for a pregnant woman that may increase vulnerability to emotional disturbances such as depression, anxiety, and stress (DAS).^1^, ^2^ The pathophysiology of DAS in pregnant women is complex and is not fully understood; however, the role of estrogen, progesterone, and cortisol, the three hormones with large psychoactive effects cannot be ignored. The level of these three hormones increase during pregnancy reaching their peaks at the end of pregnancy.^3^ Although mental health is an essential component of a women's reproductive health, it is often neglected; and antenatal psychological morbidities are frequently undetected and untreated.^2^, ^4^

Evidence suggests that many women experience psychological disorders during pregnancy. On a global basis, in normal circumstances, around 10% of pregnant women experience psychological disorders, mainly depression.^5^ The prevalence of depression among pregnant women in high‐income countries range between 7% and 15%; however, it ranges between 19% and 25% in low‐ and middle‐income countries.^6^ In South Asian region, the pooled prevalence of depression reaches to 24.7%.^7^ In individual countries, a community‐based study in India revealed that the prevalence of DAS among pregnant women was 25.5%, 63.0%, and 23.0%, respectively.^8^ Another study conducted in Saudi Arabia reported a prevalence of 26.8% and 23.6% for depression and anxiety, respectively.^9^ However, in another study conducted in Pakistan, 81.0% of pregnant women were depressed, of which 35.7% was mild, 29.0% was moderate, 11.0% was severe, and 5.3% was very severe.^10^ The considerable difference in the prevalence of psychological disorders among pregnant women in different countries may be due to several factors including differences in people's sociodemographic status, disintegration of mental health in antenatal care, and stigma.^11^

Higher prevalence of DAS has been found to be associated with mothers' young age, lower educational level, lower socioeconomic status, women's unemployment, unplanned pregnancy, low number of parity and gravida,^10^, ^12^, ^13^ history of miscarriage and failed pregnancies,^9^, ^10^ history of health complication during previous pregnancies,^14^ physical, sexual and verbal abuse,^12^, ^15^ and a lack of family and partner support.^10^, ^12^, ^16^

Before this study, there has been no published work regarding the prevalence of DAS among pregnant women in Afghanistan. This study aims to determine the prevalence and associated factors of these psychiatric disorders during pregnancy in women living in Herat province of Afghanistan.

## 2 MATERIALS AND METHODS

### 2.1 Design and setting

This hospital‐based cross‐sectional study conducted between July and November 2019 on pregnant women attending the antenatal clinic of Herat Razaei Maternity Hospital, the only public sector hospital providing tertiary Obs/Gyn care services for the residents of Herat city in western Afghanistan.

### 2.2 Eligibility criteria

The eligibility criteria included Dari‐speaking women aged 15–49 years old, living in Herat city, having a confirmed pregnancy with no medical or obstetric complications in the present pregnancy, and no history of previous psychological disorders.

### 2.3 Target population

In 2019, Herat city was home to 556,205 people, of whom 278,859 (50.1%) were female.^17^ According to National Statistics and Information Authority of the Islamic Republic of Afghanistan, 44.31% of Herat population were between the age of 15 and 49 years, bringing the total number of women in this age category to 123,563. In a study conducted by Herat University in 2019, the ratio of pregnancy among Herati women was estimated to be 11.94%.^18^ Therefore, the total number of target population of this study came to 14,754.

### 2.4 Study participants

Sample size was estimated online (http://www.raosoft.com/samplesize.html), using the following formula:

x=Z(c/100)2r(100‐r).

n=Nx/((N‐1)E2+x).

E=Sqrt[(N‐n)x/n(N‐1)].

In this formula, *Z* is the critical value, *c* is the confidence level, *r* is the fraction of responses, *n* is the estimated sample size, *N* is the population size, and *E* is the margin of error. Using a 5% *E* and 99% *c*, the minimum *n* was estimated 635. To compensate for any possible incorrect or incomplete responses, we added 10% to the estimated *n*; therefore, a total of 699 pregnant women were initially included in this study. Participants of this study were selected from the target population using a convenience sampling strategy.

### 2.5 Data collection

Data was collected using paper‐based structured questionnaires by six final‐grade medical students and regularly checked by two clinical psychologists. The interview process involved the use of a questionnaire that collected data on participant's age, number of children with congenital abnormalities, education level, employment, family economic status, parity, gestational age, planning of current pregnancy, pregnancy gap, nutritional and health status, addiction to drug, family and husband support.

In addition, the validated Dari‐translated version of 42‐item depression, anxiety, and stress scale (DASS‐42) was used.^19^ The Dari‐translated version of DASS‐42 is a valid and reliable tool for the identification and measurement of DAS. It has a good validation with Cronbach's alpha values of 0.888 for Depression, 0.866 for anxiety and 0.833 for stress.^19^ Diagnosis and categorization of DAS were performed according to DASS‐42 guideline.^20^

### 2.6 Statistical analyses

Statistical analyses were performed using IBM SPSS Statistics (version 27). Categorical variables were presented as numbers and percentages. Continuous variables with normal distribution were reported as mean ± standard deviation, those with nonnormal distribution were presented as median and interquartile range (IQR). A 2‐sided *χ* ^2^ test was used to test for association between categorical data. In bivariate regression analysis, sociodemographic characteristics with a *p* < 0.200 were included in the model. Forward likelihood ratio was used to assess the strength of association between sociodemographic variables and DAS; independently. Significance value, odds ratio and 95% confidence interval (95% CI) were reported for each prediction. The significance level was set to 0.05 and the CI to 95% in all statistical analyses.

## 3 ETHICAL CONSIDERATION

The study protocol was approved by Human Ethics Committee of Herat University (approval number #190401). Data were acquired from each participant by means of interview, after ensuring about privacy and confidentiality of information and obtaining a written informed consent.

## 4 RESULTS

### 4.1 Sociodemographic characteristics of study participants

Of the 699 questionnaires collected, eight had missing or incomplete responses, and were excluded from the study. Therefore, a total of 691 pregnant women aged between 15 and 49 (26.17 ± 6.06) years were included in the study. Sociodemographic characteristics of study participants are displayed in Table 1. Of all participants, 374 (54.1%) aged between 15 and 25 years, 475 (68.7%) were multipara, 668 (96.7%) had no experience of having kids with congenital abnormalities, 501 (72.5%) had a planned pregnancy, 458 (66.3%) had a good health status, 397 (57.5%) had a good nutritional status, 668 (96.7%) had no addiction, 506 (73.2%) were literate, 569 (82.3%) were unemployed and 410 (59.3%) had an average household economy. The median (IQR) age of participants at marriage was 18.0 (16.0–20.0) years, and the median (IQR) duration of marriage among participants was 5.0 (2.0–11.0) years.

**Table 1:** Sociodemographic characteristics of study participants.

Variables	Number	Percentage
Age		
15–25	374	54.1
26–35	260	37.6
>35	57	8.2
Parity		
First	216	31.3
Multiple	475	68.7
Abnormal kids		
Yes	23	3.3
No	668	96.7
Gestational age		
First trimester	155	22.4
Second trimester	159	23.0
Third trimester	326	47.2
Current pregnancy		
Planned	501	72.5
Unplanned	190	27.5
Mother's health		
Good	458	66.3
Average	175	25.3
Poor	58	8.4
Mother's nutrition		
Good	397	57.5
Fair	243	35.2
Poor	51	7.4
Addiction		
Yes	23	3.3
No	668	96.7
Family support		
Yes	624	90.3
No	67	9.7
Husband support		
Yes	619	89.6
No	72	10.4
Women's Education		
Illiterate	185	26.8
Literate	506	73.2
Women's employment		
Unemployed	569	82.3
Employed	122	17.7
Family economy		
Good	182	26.3
Average	410	59.3
Poor	99	14.3

### 4.2 Prevalence of depression, anxiety, and stress

Of all participants in this study, 208 (30.1%) had a moderate to extremely severe depression, 188 (27.2%) had a moderate to extremely severe anxiety, and 358 (51.8%) had a moderate to extremely severe stress. Table 2 shows the level of these mental illnesses among study participants. The median (IQR) of DAS among participants were 7.0 (1.4–15.4), 12.0 (6.0–20.0), and 18.2 (9.8–28.0), respectively.

**Table 2:** Prevalence and severity of mental illnesses among study participants.

Mental illnesses	No symptoms *n* (%)	Mild *n* (%)	Moderate *n* (%)	Severe *n* (%)	Extremely severe *n* (%)	IQR
Depression	395 (57.2)	88 (12.7)	102 (14.8)	44 (6.4)	62 (9.0)	1.4–15.4
Anxiety	407 (58.9)	96 (13.9)	130 (18.8)	50 (7,2)	8 (1.2)	6.0–20.0
Stress	280 (40.5)	53 (7.7)	143 (20.7)	138 (20.0)	77 (11.1)	9.8–28.0

Abbreviations: IQR, interquartile range; *n*, number of participants.

**Figure 1:**
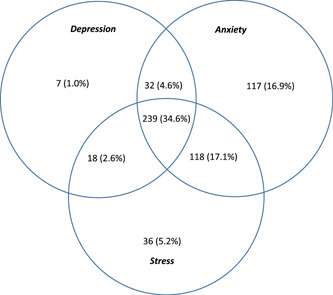
Numbers and percentages of study participants suffering from depression, anxiety, and stress in this study.^3^, ^20^

Of all participants in this study, 124 (17.9%) had no symptoms of DAS, while 239 (34.6%) had DAS, of which 20 (2.9%) lived with extremely severe DAS. Figure 1 shows the overlapping prevalence of DAS among study participants.

### 4.3 Association of sociodemographic characteristics of study participants with depression, anxiety, and stress

All sociodemographic variables included in this study (except gestational age) were significantly associated with depression and anxiety. Concerning stress, age, parity, gestational age, and women's addiction were insignificantly associated with stress. Table 3 shows the association between sociodemographic variables with DAS in this study.

**Table 3:** Association between sociodemographic variables and mental illnesses in this study.

Variables	Total numbers	Depression (*n* [%])	Anxiety (*n* [%])	Stress (*n* [%])
Age				
15–25	374	144 (38.5%)	132 (35.3%)	212 (56.7%)
26–35	260	123 (47.3%)	121 (46.5%)	165 (63.5%)
>35	57	29 (50.9%)	31 (54.4%)	34 (59.6%)
*p* Value		0.039	0.002	0.232
Parity				
First	216	77 (35.6%)	74 (34.3%)	118 (54.6%)
Multiple	475	219 (46.1%)	210 (44.2%)	293 (61.7%)
*p* Value		0.010	0.014	0.080
Abnormal kids				
Yes	23	14 (60.9%)	16 (69.6)	21 (91.3)
No	668	282 (42.2%)	268 (40.1)	390 (58.4)
*p* Value		0.075	0.005	0.002
Gestational age				
First trimester	155	69 (44.5)	54 (34.8)	79 (51.0)
Second trimester	159	65 (40.9)	58 (36.5)	101 (63.5)
Third trimester	326	139 (42.6)	149 (56.7)	198 (60.7)
Postterm	51	23 (45.1)	23 (45.1)	33 (64.7)
*p* Value		0.910	0.069	0.089
Planning of current pregnancy				
Planned	501	188 (37.5)	178 (35.5)	284 (56.7)
Unplanned	190	108 (56.8)	106 (55.8)	127 (66.8)
*p* Value		<0.001	<0.001	0.015
Mother's health				
Good	458	166 (36.2)	158 (34.5)	245 (53.5)
Average	175	94 (53.7)	90 (51.4)	120 (68.6)
Poor	58	36 (62.1)	36 (62.1)	46 (79.3)
*p* Value		<0.001	<0.001	<0.001
Husband's health				
Good	529	205 (38.8)	201 (38.0)	303 (57.3)
Average	119	58 (48.7)	53 (44.5)	73 (61.3)
Poor	43	33 (76.7)	30 (69.8)	35 (81.4)
*p* Value		<0.001	<0.001	0.007
Mother's nutrition				
Good	397	143 (36.0)	132 (33.2)	217 (54.7)
Fair	243	118 (48.6)	120 (49.4)	156 (64.2)
Poor	51	35 (68.1)	32 (62.7)	38 (74.5)
*p* Value		<0.001	<0.001	0.004
Mother's addiction				
Yes	23	16 (69.6)	15 (65.2)	17 (73.9)
No	668	280 (41.9)	269 (40.3)	394 (59.0)
*p* Value		0.008	0.017	0.152
Family support				
Yes	624	245 (39.3)	240 (38.5)	361 (57.9)
No	67	51 (76.1)	44 (65.7)	50 (74.6)
*p* Value		<0.001	<0.001	0.008
Husband's support				
Yes	619	244 (39.4)	234 (37.8)	355 (57.4)
No	72	52 (72.2)	50 (69.4)	56 (77.8)
*p* Value		<0.001	<0.001	0.001
Mother's education				
Illiterate	185	98 (53.0)	105 (56.8)	123 (66.5)
Literate	506	198 (39.1)	179 (35.4)	288 (56.9)
*p* Value		0.001	<0.001	0.023
Mother's employment				
Unemployed	569	258 (45.3)	248 (43.6)	349 (61.3)
Employed	122	38 (31.1)	36 (29.5)	62 (50.8)
*p* Value		0.004	0.004	0.032
Family economy				
Poor	99	63 (63.6)	64 (64.6)	66 (66.7)
Average	410	183 (44.6)	17 (41.5)	252 (61.5)
Good	182	50 (27.5)	50 (27.5)	93 (51.1)
*p* Value		<0.001	<0.001	0.017

In a bivariate regression analysis, poor and fair women's health, unplanned pregnancy, lack of husband's support, lack of family support, and poor and fair family economy were found to significantly increase the development of depression (Table 4), while poor and fair women's health, unplanned pregnancy, lack of husband's support, women's illiteracy; and poor and fair family economy were predictors of anxiety (Table 5). For stress, the significant predictors were found to be poor and fair women's health, history of having children with congenital abnormalities, and lack of husband's support (Table 6).

**Table 4:** Regression analysis model of sociodemographic factors predicting depression.

Variables	B	SE	Wald	*df*	*p* Value	Exp (B)	95% CI for Exp (B)	
							Lower	Upper
Women's health (Reference: good)								
Poor	0.469	0.191	6.008	1	0.014	1.598	1.099	2.325
Fair	0.700	0.309	5.124	1	0.024	2.013	1.098	3.691
Planning of current pregnancy (Reference: planned)								
Unplanned	0.456	0.188	5.887	1	0.017	1.577	1.092	2.279
Husband's support (Reference: yes)								
No	0.825	0.301	7.538	1	0.006	2.283	1.266	4.115
Family support (Reference: Yes)								
No	1.012	0.322	9.871	1	0.002	2.752	1.463	5.174
Family economy (Reference: good)								
Poor	1.096	0.283	15.039	2	<0.001	2.992	1.720	5.207
Fair	0.632	0.199	10.053	1	0.002	1.882	1.273	2.782

Abbreviations: B, unstandardized odds ratio; CI, confidence interval; *df*, degree of freedom; Exp (B), odds ratio; *p*, probability value; SE, standard error.

**Table 5:** Regression analysis model of sociodemographic factors predicting anxiety.

Variables	B	SE	Wald	*df*	*p* Value	Exp (B)	95% CI for Exp (B)	
							Lower	Upper
Women's health (Reference: good)								
Poor	0.430	0.192	5.024	1	0.025	1.538	1.055	2.240
Fair	0.798	0307	6.735	1	0.009	2.220	1.216	4.055
Planning of current pregnancy (Reference: planned)								
Unplanned	0.521	0.186	7.827	1	0.005	1.683	1.1691	2.425
Husband's support (Reference: yes)								
No	0.971	0.285	11.641	1	0.001	2.640	1.512	4.611
Women's education (Reference: literate)								
Illiterate	0.515	0.192	7.234	1	0.007	1.674	1.150	2.438
Family economy (Reference: good)								
Poor	1.026	0.290	12.518	1	<0.001	2.790	1.580	4.925
Fair	0.477	0.200	5.664	1	0.017	1.611	1.088	2.386

Abbreviations: B, unstandardized odds ratio; CI, confidence interval; *df*, degree of freedom; Exp (B), odds ratio; *p*, probability value; SE, standard error.

**Table 6:** Regression analysis model of sociodemographic factors predicting stress.

Variables	B	SE	Wald	*df*	*p* Value	Exp (B)	95% CI for Exp (B)	
							Lower	Upper
Women's health (Reference: good)								
Poor	0.573	0.191	9.015	1	0.003	1.773	1.220	2.577
Fair	1.106	0.342	10.460	1	0.001	3.021	1.546	5.904
Having children with congenital abnormalities (Reference: No)								
Yes	1.808	0.752	5.776	1	0.016	6.096	1.396	26.623
Husband's support (Reference: yes)								
No	0.757	0.303	6.244	1	0.012	2.131	1.177	3.857

Abbreviations: B, unstandardized odds ratio; CI, confidence interval; *df*, degree of freedom; Exp (B), odds ratio; *p*, probability value; SE, standard error.

## 5 DISCUSSION

This study assessed the prevalence and associated factors of DAS among pregnant women in Herat, Afghanistan.

The prevalence of depression in this study was 42.8%, which is similar to results reported from Nigeria (45.2%).^21^ However, our results differ from findings of similar studies conducted among pregnant women in India (25.5%), Iran (31.7%), and China (36.2%).^8^, ^22^, ^23^ Of all participants in this study, 12.7% suffered from mild depression, while 30.2% lived with moderate to extremely severe depression. This contradicts with a study conducted in China, in which 25.8% of study participants reported to have had mild depression, and 10.4% have lived with moderate to severe depression.

We found that over 40% of our participants suffered from anxiety. This is much less than results reported from India and Nigeria, in which two‐thirds of pregnant women in both studies experienced anxiety.^8^, ^21^ However, similar studies from Iran, Italy, and China reported that 32.5%, 32.0%, and 26.9% of pregnant women lived with anxiety, respectively.^22^, ^23^, ^24^ The mild and moderate to extremely severe anxiety rates in this study were 13.9% and 27.2%, respectively. This is in line with a study from Nigeria that reported 14.7% and 22.8% mild and extremely severe anxiety among study participants, respectively^21^; however, it is unlike the results reported from China in which 22.4% and 4.5% of study participants had a mild and moderate to severe anxiety, respectively.^22^

In our study, the prevalence of stress was 59.5%. This is in line with the results reported from China that at least 60.4% of pregnant women experienced stress.^22^ The prevalence of mild and moderate to extremely severe stress in our study was 7.7% and 51.8%, respectively. This is in accordance with another study from Nigeria, which reported 5.5% mild, and 51.3% moderate to extremely severe stress among study participants.^21^ The reason behind a more severe DAS among our study participants, compared to many similar studies conducted elsewhere, may attribute to several other factors such as ongoing arm‐conflict, higher levels of civil and family violence, lower socioeconomic, general health and nutritional status of people in Herat.

Our results indicate that gestational age was not significantly associated with DAS (*p* > 0.05). Similar findings were reported from the United Kingdom, Slovenia, and Malaysia in which an insignificant association was found between DAS with gestational age.^1^, ^14^, ^25^ However, two studies from Iran found a significant association between mental disorders and gestational age among pregnant women included in the study.^23^, ^26^

We also found that unplanned (unwanted) pregnancy was significantly associated with DAS. Similar findings were reported from Pakistan, Iran, and India.^12^, ^15^, ^23^, ^26^, ^27^, ^28^ This study also revealed that parity was significantly associated with depression and anxiety and not with stress. Similar studies also reported a significant association between parity with depression and anxiety,^2^, ^15^, ^28^ only with depression,^21^ and only with anxiety.^29^

We also found that family support, especially husband's support during pregnancy was significantly associated with a lower level of DAS. A study in China also revealed that low social support was associated with higher levels of depression.^30^ Other studies from Pakistan, Malaysia, and Iran found a link between family violence with depression and anxiety,^15^, ^25^ and only with anxiety.^29^

This study revealed a significant association between women's education and level of DAS. Studies from China and Iran also reported similar findings.^15^, ^22^, ^29^ However, an insignificant association between women's level of education with depression and stress was reported from Iran.^26^

This study revealed that women's poor health and nutrition status were significantly associated with higher levels of DAS, which is consistent with the findings of a study from Canada.^27^ However, another study from China reported a significant association between previous medical history with anxiety and not with depression and stress.^30^ This reveals that pre‐existing health problems, and poor nutritional status are among the influencing factors in the development of mental disorders during pregnancy.

Poor self‐perceived family economy was also associated with higher levels of DAS, in this study. Similar findings were reported from Iran, Saudi Arabia, Malaysia, and Bangladesh.^9^, ^25^, ^26^, ^31^

## 6 LIMITATIONS

The current study has a few limitations. First, the exclusion of nonpregnant women in this study failed to provide data for comparison. Hence, we could not be certain if the magnitude of DAS among pregnant women in this study is different from that of general population. Second, diagnosis of mental disorders in this study was merely based on DASS‐42 questionnaire; the gold standard method of diagnosis of these illnesses is through physical and clinical examination. Finally, this study only included Dari‐speaking pregnant women, because the study instrument (DASS‐42 questionnaire) was only validated in Dari so far.

## 7 RECOMMENDATIONS

As DASS‐42 questionnaire is only validated in Dari‐speaking Afghan population, translation and validation of this instrument in other most widely spoken languages in Afghanistan is highly recommended. Furthermore, considering the high prevalence of DAS among pregnant women in Herat, mental health interventions such as cognitive behavior therapy and psychological counseling, either online or in person, is recommended to prevent or reduce the sequalae of these mental disorders among pregnant women and their families in the region.

## 8 CONCLUSION

The prevalence of DAS is very high among pregnant women in Herat. Pregnancy planning, women's health, women's education, family support, having children with congenital abnormalities, and family economy are the risk factors for developing mental disorders among pregnant women. Considering scientific evidence on high prevalence and associated factors of DAS among pregnant women, policymakers, public health authorities, and medical practitioners must devote significant attention in reducing the magnitude of these mental disorders and/or reducing their impact on women, their families, and society.

## AUTHOR CONTRIBUTIONS

**Aziz‐ur‐Rahman Niazi**: Conceptualization; data curation; formal analysis; methodology; supervision; writing—original draft; writing—review and editing. **Mina Alekozay**: Conceptualization; data curation; formal analysis; methodology; writing—original draft; writing—review and editing. **Khadejah Osmani**: Conceptualization; methodology; writing—original draft; writing—review and editing. **Abdul Fattah Najm**: Conceptualization; methodology; writing—original draft; writing—review and editing.

## CONFLICT OF INTEREST STATEMENT

The authors declare no conflict of interest.

## TRANSPARENCY STATEMENT

The lead author Aziz‐ur‐Rahman Niazi affirms that this manuscript is an honest, accurate, and transparent account of the study being reported; that no important aspects of the study have been omitted; and that any discrepancies from the study as planned (and, if relevant, registered) have been explained.

## Data Availability

The data that support the findings of this study are available from the corresponding author upon reasonable request.
